# *Burkholderia cepacia,* cystic fibrosis and outcomes following lung transplantation: experiences from a single center in Brazil

**DOI:** 10.6061/clinics/2018/e166

**Published:** 2018-03-07

**Authors:** Danila de Souza Carraro, Rafael Medeiros Carraro, Silvia Vidal Campos, Leandro Ryuchi Iuamoto, Karina Andrighetti de Oliveira Braga, Lea Campos de Oliveira, Ester Cerdeira Sabino, Flavia Rossi, Paulo Manuel Pêgo-Fernandes

**Affiliations:** I Divisao de Cirurgia Toracica, Instituto do Coracao (InCor), Hospital das Clinicas HCFMUSP, Faculdade de Medicina, Universidade de Sao Paulo, Sao Paulo, SP, BR; II Divisao de Pneumologia, Instituto do Coracao (InCor), Hospital das Clinicas HCFMUSP, Faculdade de Medicina, Universidade de Sao Paulo, Sao Paulo, SP, BR; III Cardiopneumologia, Faculdade de Medicina FMUSP, Universidade de Sao Paulo, Sao Paulo, SP, BR; IV Laboratorio de Investigacao Medica (LIM3), Faculdade de Medicina FMUSP, Universidade de Sao Paulo, Sao Paulo, SP, BR; V Departamento de Biologia Molecular Divisao de Sorologia, Fundacao Pro Sangue Hemocentro de Sao Paulo, Secretaria de Saude do Estado de São Paulo, São Paulo, SP, BR; VI Microbiologia, Divisao de Laboratorio Central, Hospital das Clinicas HCFMUSP, Faculdade de Medicina, Universidade de Sao Paulo, Sao Paulo, SP, BR

**Keywords:** Lung Transplantation, Cystic Fibrosis, *Burkholderia Cepacia* Complex, *Burkholderia cenocepacia*, Prognosis

## Abstract

**OBJECTIVES:**

To evaluate the impact of *Burkholderia cepacia* complex colonization in cystic fibrosis patients undergoing lung transplantation.

**METHODS:**

We prospectively analyzed clinical data and respiratory tract samples (sputum and bronchoalveolar lavage) collected from suppurative lung disease patients between January 2008 and November 2013. We also subtyped different *Burkholderia cepacia* complex genotypes via DNA sequencing using primers against the recA gene in samples collected between January 2012 and November 2013.

**RESULTS:**

From 2008 to 2013, 34 lung transplants were performed on cystic fibrosis patients at our center. *Burkholderia cepacia* complex was detected in 13 of the 34 (38.2%) patients. Seven of the 13 (53%) strains were subjected to genotype analysis, from which three strains of *B. metallica* and four strains of *B. cenocepacia* were identified. The mortality rate was 1/13 (7.6%), and this death was not related to *B. cepacia* infection.

**CONCLUSION:**

The results of our study suggest that colonization by *B. cepacia* complex and even *B. cenocepacia* in patients with cystic fibrosis should not be considered an absolute contraindication to lung transplantation in Brazilian centers.

## INTRODUCTION

Lung transplantation (LTx) can prolong the survival of and improve the quality of life for patients with reduced lung function and short life expectancy, and the procedure is used as the final therapeutic option for end-stage lung disease in more than 150 centers worldwide 1.

The number of patients on waiting lists for LTx has increased in recent years, surpassing the number of organs available for the procedure. Consequently, the waiting-list mortality rate has increased to up to 50% in some countries 2. In Brazil, in the first three months of 2015, the rate of donor-lung utilization was only 4.1%, which is much lower than the expected average of 15%. This deficiency has contributed to a waiting-list mortality rate of 10% 3.

Contraindications to transplantation such as comorbidities, poor prognosis and post-transplant complications must be considered when selecting candidates for inclusion on waiting lists 2,4.

Cystic fibrosis (CF) patients are often colonized by multiple bacterial species. Despite its low prevalence, *Burkholderia cepacia* complex (BCC) colonization has been associated with a poorer prognosis after LTx. This bacterial complex includes 20 genotypically distinct species or “genomovars” 5-9, and the most severe infections seem to be related to genomovar III (*B. cenocepacia*) 10,11.

Recent studies have confirmed an association between the presence of genomovar III and reduced survival after LTx 12-14. Because of this relationship, some centers have begun to exclude patients colonized by *B. cenocepacia* from waiting lists for LTx.

In our center, over 50% of bilateral transplants are performed in patients with suppurative disease, 26% of whom are CF patients. The transplants have resulted in very good outcomes, with an overall survival of 55.2% after 5 years 15. Furthermore, unlike other groups, we have not observed an increased morbidity in CF patients colonized by BCC. Therefore, the aim of the current study is to describe the incidence of preoperative colonization by BCC in candidates for LTx at our lung transplant center and to analyze the impact of this colonization on clinical outcomes after LTx.

## METHODS

The current study used a prospective cohort design. Clinical data and microbiology results were collected for all patients (adults and children) with CF who underwent LTx between January 2008 and November 2013 at the Heart Institute of Sao Paulo Medical School (InCor – HCFMUSP). The two ethics committees at this institute approved the research.

Clinical data were prospectively collected to compare clinical outcomes after LTx between CF patients colonized or not colonized by *Burkholderia cepacia* complex.

The surgical and clinical protocols are described below.

**Surgery technique:** All included patients underwent bilateral LTx via a bilateral thoracotomy (clamshell incision) with insertion of two pleural drainage tubes on each side of the thorax.

**Immunosuppression protocol:** For induction of immunosuppressive treatment, all patients received methylprednisolone (500 mg during surgery) and basiliximab (20 mg during surgery and on day 4). For maintenance immunosuppression therapy, tacrolimus (0.15 mg/kg/d), azathioprine (2 mg/kg/d) and prednisone (0.5 mg/kg/d) were used for 1 month with dose reductions over the following months. After November 2010, our initial immunosuppressive protocol was changed to cyclosporine (5 mg/kg bid), mycophenolate sodium (10 mg/kg bid up to 1440 mg/d) and prednisone (0.5 mg/kg/d) for all CF patients due to the lower diabetogenic profile than tacrolimus-based schemes.

**Monitoring and treatment of acute rejection episodes:** Surveillance fiber optic bronchoscopy was performed in the second and sixth weeks after transplantation and then in months 3, 6, 9 and 12 or when clinically indicated. Acute rejection episodes were always treated with a 3-day pulse of methylprednisolone (10 mg/kg/d up to 1 g/d) when biopsies were graded as A2 or higher according to the International Society of Heart and Lung Transplant revision of the 1996 working formulation for the standardization of nomenclature in the diagnosis of lung rejection 16. Perivascular infiltrates graded as A1 and bronchiolar lymphocytic infiltrates had individualized therapeutic strategies.

**Bacterial prophylaxis:** Antibiotics were chosen based on pre-transplant culture sputum results. All patients colonized with BCC were treated with at least two drugs (trimethoprim-sulfamethoxazole and meropenem) until intraoperative cultures of bronchial aspiration and bronchoalveolar lavage samples were available. Inhaled antibiotics (tobramycin or colistin) were also used for all CF patients. Sputum culture samples were collected from all patients on the waiting list every 3 to 6 months and in cases of acute exacerbation.

**Microbiology:** Respiratory tract samples were plated on selective agar for *B. cepacia* and incubated at 37°C for 72 hours (Oxoid, USA). Isolated strains were identified using a Vitek automated system (bioMérieuxVitek Inc., Marcy-L'Etoile, France); specifically, the strains were subjected to sensitivity testing against meropenem, ceftazidime, and trimethoprim-sulfamethoxazole. This technique was performed in accordance with the guidelines recommended by the Clinical and Laboratory Standards Institute (CLSI 2010) 17.

**Molecular biology:** Genotyping was performed using capillary electrophoresis sequencing. Molecular analysis of *B. cepacia* genotypes was performed using DNA sequencing primers against the recA11 18 gene. After the amplification of DNA fragments, automatic sequencing, alignment and phylogenetic analysis were performed on the sequences. Evolutionary relationships between recA genes were determined through data analysis using the software Molecular Biology. DNA extraction was performed on positive samples using a QIAamp® DNA minikit (Qiagen, Germany). The partial recA gene of the *Burkholderia* genome was amplified as reported by Payne et al (2005). Sequencing reactions were performed using a Bigdye Terminator v.3.1 Cycle Sequencing Ready Reaction Kit on an ABI PRISMDye Deoxy Terminator Automatic DNA sequencer (Applied Biosystems Inc., Foster City, CA, USA).

**Phylogenetic analysis:** Sequences were aligned using Sequencer version 4.1.4. A maximum likelihood (ML) phylogenetic tree was generated. The same model was also used to estimate maximum a posteriori (MAP) trees using a Bayesian Markov Chain Monte Carlo (MCMC) method.

**Statistical analysis:** All analyses were performed using SPSS software, version 13.0. For univariate analysis and to estimate the independent effects of variables, a Cox proportional hazards model was used with 95% confidence intervals. *P* < 0.05 was considered statistically significant. The Kaplan-Meier method was used to generate survival curves. Nine independent categorical variables were analyzed via univariate Cox regression analysis: age, sex, body mass index (BMI), colonization by BCC, primary graft dysfunction (PGD), acute rejection episodes, chronic rejection, days in intensive care unit (ICU) and days until hospital discharge after transplantation.

## RESULTS

Between 2008 and 2013, 130 lung transplants were performed at our institution. Overall, 62 (47.6%) transplantations were performed in patients with suppurative lung disease, and 34 (26.1% of the total) of these patients had CF.

BCC was identified in 13 patients with CF. Among these, seven strains of BCC were genetically analyzed: 3 were identified as strains of *Burkholderia metallica* and 4 as strains of *Burkholderia cenocepacia*. Regarding the prevalence of colonization with other bacteria, 55% (19/34) of the CF patients were positive for *Staphylococcus aureus* and 82% (28/34) for *Pseudomonas aeruginosa*.

### *Burkholderia cepacia* complex infection and clinical outcomes

During follow-up, the mortality rate was 17.6% (6/34). Among the six CF patients who died after LTx, only one was previously colonized by BCC. However, this patient's death was attributed to hospital-acquired pneumonia and septic shock due to multi-drug-resistant *Acinetobacter baumannii*. Of note, no BCC-positive cultures were identified among the samples collected during post-transplant follow-up.

**Figure 1:**
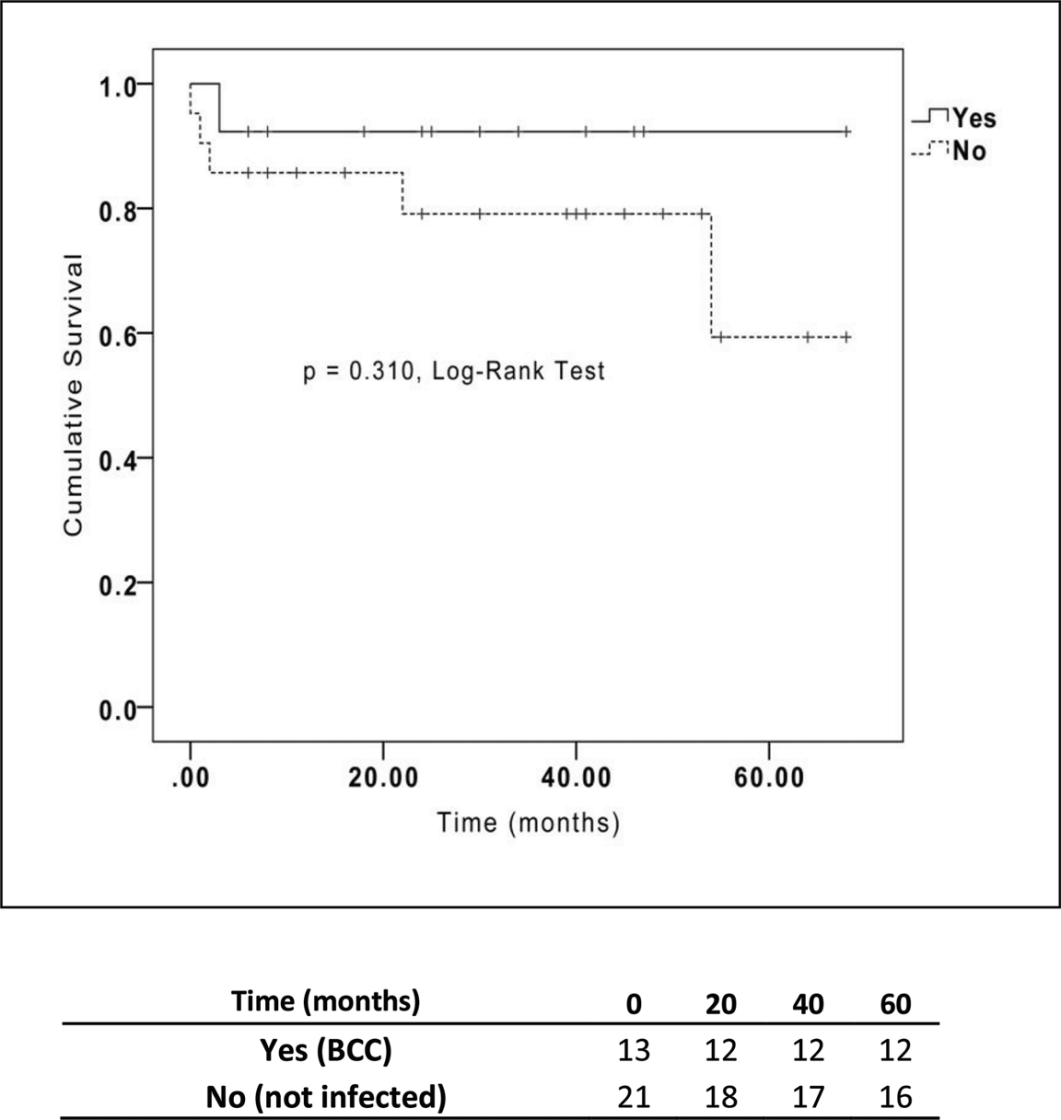
Kaplan-Meier survival curves for cystic fibrosis patients who underwent lung transplantation classified by *Burkholderia cepacia* complex infection status.

**Figure 2:**
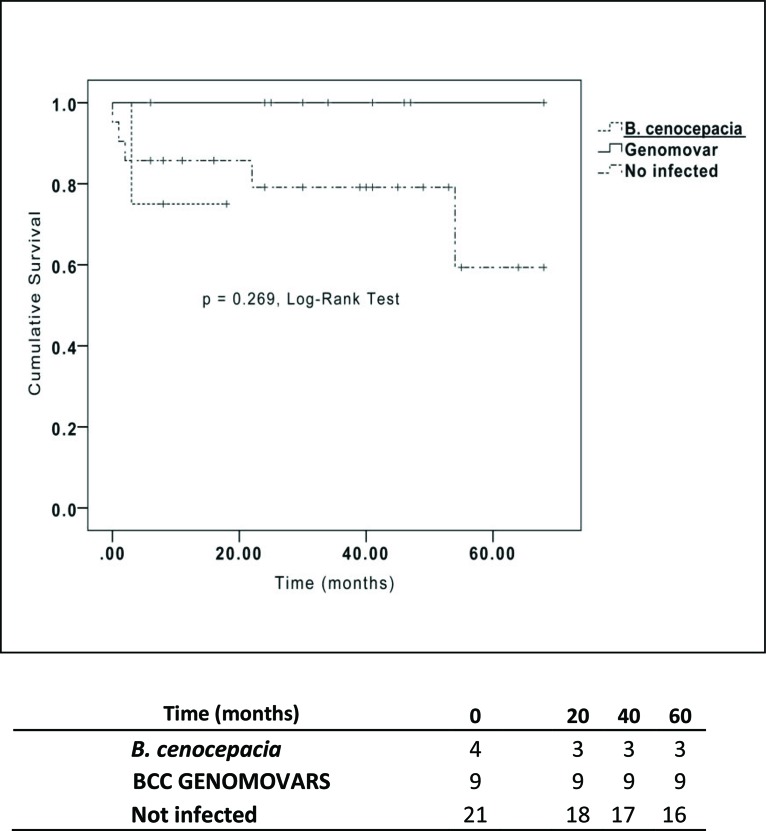
Kaplan-Meier Survival curves for patients with cystic fibrosis who underwent lung transplantation classified by *B. cenocepacia* infection status.

The characteristics and clinical outcomes of the CF patients included in our study are summarized in Table 1. The results from the univariate analysis are shown in Table 2. BCC-colonized and non-BCC-colonized patients showed no differences in mortality rate, length of ICU stay, length of hospital stay, primary graft dysfunction, acute rejection or allograft chronic dysfunction (Table 2 and Figure 1). Of special interest, whether a patient was colonized with the BCC strains *B. metallica* or *B. cenocepacia* did not impact the mortality rate after LTx (Figure 2).

## DISCUSSION

LTx recipients should be rationally selected based on considerations of the risk of mortality without a transplant, the chance of patient survival after transplantation and the expected time on the waiting list 2,3. Identifying and excluding possible causes of increased post-transplant mortality can optimize LTx survival outcomes and even reduce the waiting list length. Indeed, some centers have found that both short- and long-term survival are significantly lower for patients preoperatively infected with *B. cenocepacia*, leading to the difficult decision to stop listing these patients 12-14.

CF is the third most common indication for lung transplantation. Interestingly, despite that CF involves multiple organs and has characteristics that could aggravate morbidity and mortality after LTx, CF patients have shown the best survival rates after transplantation compared to all other indications 19.

In contrast with previous investigations of CF patients and BCC colonization, our study found no difference in post-LTx survival between patients colonized and not colonized with BCC, even in a separate analysis of *B. cenocepacia* colonization. Indeed, only one death occurred among the *B. cenocepacia*-colonized patients, and the cause of death was attributed to hospital-acquired *Acinetobacter baumannii* infection. Thus, this case did not indicate that BCC increased the risk of septic complications. No evidence of BCC colonization was identified in any culture sample after the transplant procedure.

Literature regarding the impact of BCC colonization on transplant outcomes is controversial. Older studies have shown poor post-transplant survival of patients colonized by BCC, with survival rates of 50% and 83% being reported for BCC-infected and non-infected patients, respectively (*p* = 0.006). However, virulence markers and specific BCC subtypes (e.g., *B. cenocepacia* - genomovar III) have been directly related to worse prognosis after transplantation 11. More recently, The Freeman Hospital (UK) LTx group examined a cohort of 22 BCC-infected CF patients, including patients infected with *B. cenocepacia* and other BCC genomovars, and found no significant decrease in survival 14; however, the specific mortality of patients infected with genomovar III (*B. cenocepacia*) was excessively high (75%). Lung transplant centers in North America and France have also published data on this topic, with similar conclusions to our study, namely, that colonization by BCC does not impact post-LTx survival and is therefore not a reason to exclude colonized CF patients from LTx waiting lists. However, careful screening of BCC-infected patients is recommended, and healthcare providers should be aware that post-transplant mortality among BCC-infected patients with CF varies by infecting species. For example, *B. cenocepacia* was not associated with an unacceptably high risk of a fatal outcome after LTx, although a relationship with poorer long-term survival was found 20,21.

Regarding our cohort, the small number of patients, especially those colonized with *B. cenocepacia* (only 4 patients), is certainly a limitation of the study. However, this is the only study conducted to date examining a Brazilian population that has matched BCC genomovar findings with good clinical outcomes.

There was a difference in mortality related to *B. cenocepacia* infection in our study compared to previous reports, and this discrepancy may be related to host factors. The relationship between the genetic determinants in the population, the host immune response and the interaction between *B. cenocepacia* and the respiratory microbiome of CF patients from Brazil has never been studied. It should be noted that all *B. cenocepacia* strains may not exhibit the same behavior and that the strain identified in our cohort was potentially less virulent than those present in other centers.

Although our sample size was small, we did not find that CF patients colonized by BCC exhibited a worse prognosis after LTx, even patients infected with *B. metallica* or *B. cenocepacia*. Based on these results, we suggest that BCC colonization (even with genomovar III) should not be a contraindication for LTx in CF patients in Brazil. The potential that both CF patients and *B. cenocepacia* strains exhibit different characteristics depending on world location is intriguing, and a clearer understanding of these differences should be sought moving forward.

## AUTHOR CONTRIBUTIONS

Carraro DS and Carraro RM were responsible for the data collection and analysis. Campos SV was responsible for the data analysis and manuscript review. Iuamoto LR was responsible for the data collection. Braga KA was responsible for the manuscript review. Oliveira LC was responsible for the molecular genotyping of bacterial isolates. Sabino EC and Rossi F were responsible for the analysis of respiratory culture samples. Pêgo-Fernandes PM was responsible for the final review of the manuscript.

## Figures and Tables

**Table 1:** Characteristics of the study population and distributions of clinical outcomes among subgroups.

	Not infected (n = 21)	*B. cenocepacia* (n = 4)	*B. metallica* (n = 3)	BCC No genomovar available (n = 6)
Age (yr)	22.52 ± 7.06	27 ± 11.34	26.33 ± 14.57	29.33 ± 10.38
Gender	8 F/13 M	3 F/1 M	2 F/1 M	3 F/3 M
BMI	18.66 ± 3.76	19.52 ± 5.10	19.5 ± 2.16	19.77 ± 3.95
Days of ICU stay after transplantation	11.05 ± 11.37	22.75 ± 21.76	13 ± 13.86	10.5 ± 4.50
Days of hospital stay after transplantation	55.29 ± 23.87	52.5 ± 24.12	59.67 ± 34.79	43.16 ± 16.51
Time since transplantation (mo)	36.43 ± 23.87	17.75 ± 10.90	48 ± 31.43	41.5 ± 9.09
Death (yes/no)	5	1	none	none
PGD	4	2	1	none
Chronic rejection (yes/no)	2	1	2	2
Acute rejection (yes/no)	11	3	1	5
Antibiotic sensitivity	-	1 yes/3 no	2 yes/1 no	3 yes/3 no

BMI: body mass index; ICU: intensive care unit; PGD: primary graft dysfunction.

**Table 2:** Univariate cox regression analysis.

Variable	HR	CI (95%)	*p*
Length of hospital stay after transplantation	1.017	0.970 - 1.066	0.487
Length of ICU stay after transplantation	1.045	0.993 - 1.099	0.090
BCC infection (yes/no)	0.312	0.036 - 2.674	0.288
PGD (yes/no)	0.732	0.086 - 6.271	0.776
Gender	0.459	0.083 - 2.554	0.374
BMI (≤18.5/>18.5)	2.141	0.392 - 11.726	0.379
Age (yr)	0.990	0.904 - 1.085	0.832
Chronic rejection (yes/no)	0.631	0.073 - 5.449	0.675
Acute rejection (yes/no)	0.581	0.116 - 2.898	0.507

ICU: intensive care unit; BCC: *Burkholderia cepacia* complex; PGD: primary graft dysfunction; BMI: body mass index.
